# Potential Role of Bioactive Proteins and Peptides Derived from Legumes towards Metabolic Syndrome

**DOI:** 10.3390/nu14245271

**Published:** 2022-12-10

**Authors:** Marta Garcés-Rimón, Diego Morales, Marta Miguel-Castro

**Affiliations:** 1 Grupo de Biotecnología Alimentaria, Universidad Francisco de Vitoria, 28223 Madrid, Spain; 2 Nutrigenomics Research Group, Department of Biochemistry and Biotechnology, Universitat Rovira i Virgili, 43007 Tarragona, Spain; 3 Institute of Food Science Research—CIAL (UAM+CSIC), C/Nicolas Cabrera 9, Campus de Cantoblanco, Universidad Autónoma de Madrid, 28049 Madrid, Spain

**Keywords:** legumes, bioactive peptides, antioxidant, hypocholesterolemic, antihypertensive, metabolic syndrome

## Abstract

Legumes have been widely consumed and used to isolate bioactive compounds, mainly proteins. The aim of this study was to review the beneficial actions of different legumes proteins and peptides updating the main findings that correlate legumes consumption and the effects on non-transmissible chronic diseases, specifically metabolic syndrome. An exhaustive revision of five relevant bioactivities (antioxidant, anti-inflammatory, antihypertensive, hypocholesterolemic -all of them linked to metabolic syndrome- and antitumoral) of proteins and peptides from legumes focused on isolation and purification, enzymatic hydrolysis and in vitro gastrointestinal digestion was carried out. The promising potential of bioactive hydrolysates and peptides from pulses has been demonstrated by in vitro tests. However, only a few studies validated these biological activities using animal models. No clinical trials have been carried out yet; so further research is required to elucidate their effective health implications.

## 1. Introduction

Nowadays, the link between food and health is increasingly consolidated. In the last decades, nutritional compounds are attracting much attention because, compared to drugs, they could be used as a safe and less expensive treatment for some chronic diseases and/or their complications [1]. Therefore, the interest to obtain novel “functional foods” in research is growing rapidly. “Functional foods” are defined as foods those, beyond their nutritional properties, contain biologically active components with health promoting activities and potential therapeutic use for several chronic diseases [2]. The great diversity of food bioactive compounds that can be obtained from natural sources (animal, plants, fungal, algae species, etc.) encourages the study and development of efficient technologies to isolate purified or enriched fractions from those matrices that can be potentially used to design novel or functional foods. Among the different groups of bioactive molecules, proteins and peptides should be highlighted because their health implications.

Over the last century, protein research has mainly studied the importance of essential amino acids as nutrients that must be part of the diet as a key component for the correct functioning of the human body, being involved in plenty of metabolic and physiological processes (e.g., protein synthesis, glycemia control, fat metabolism, nervous and immune system regulation, etc.). For a long time, food from animal origin has been considered the best source of functional proteins and, although the presence of proteins with biological activity has been also observed in plant sources, research has focused especially on proteins from animal matrices [3].

Bioactive peptides are inactive amino acid sequences within the precursor protein those exert certain physiological functions in the organism after their release by hydrolysis in vitro (chemical or enzymatic hydrolysis) or in vivo (gastrointestinal digestion). These molecules have been defined as “peptides with hormone- or drug-like activity” that eventually modulate physiological functions through binding interactions to specific receptors on target cells, leading to induction of physiological responses [4,5]. Since its discovery in 1979, peptides derived from food proteins have been described with different biological activities such as antioxidant, antimicrobial, opioid, immunomodulatory, antihypertensive, antidiabetic and hypocholesterolemic, among others [6,7]. Recently, there has been emerging considerable interest to identify and characterize these bioactive peptides from plant and animal origin. One of the methods most widely used for obtaining new bioactive peptides from food proteins is enzymatic hydrolysis, in which the protein sources, the type of enzyme and the degree of hydrolysis will define the peptides obtained and their biological activities [8]. Currently, there are several research groups focused on the development of hydrolysates those exhibit multifunctional properties to prevent or treat multifactorial diseases with a high prevalence in our society, such as metabolic syndrome, a chronic non-communicable disease that is one of the leading causes of death and disability worldwide. Metabolic syndrome refers to a multifunctional metabolic disorder that includes hypertension, impaired glucose tolerance, dyslipidemia and obesity, being this last one a fundamental component in the etiology of the syndrome. Furthermore, this cluster of conditions is increasingly associated with some types of cancer [1]. In this context, the beneficial effects (antioxidant, anti-inflammatory, hypocholesterolemic, antihypertensive, antitumoral) demonstrated by bioactive peptides derived from food proteins could be considered a novel strategy for the prevention and/or treatment of metabolic syndrome and related disorders.

Moreover, plants are acquiring special relevance for its use as a cheap source of proteins with biological activity. Vegetal proteins, which are mainly found in legumes (soybean, bean, pea), cereals (corn, wheat, rice), pseudocereals (buckwheat, quinoa, amaranth), seeds and nuts, are becoming especially relevant for use as a source of biologically active compounds. In addition, the United Nations Food and Agriculture Organization (FAO), has referred to plants as a rich source of high-quality proteins for human consumption, highlighting their abundance in nature, their nutritional value and the absence of cholesterol, as advantages. It also points out that products based on vegetal proteins, could also be consumed by vegans or people who avoid the consumption of animal products for religious/health reasons or to improve sustainability with regimens less dependent on meat proteins, contributing to meet the nutritional needs of world population [9,10].

Numerous studies about bioactive peptides have focused on vegetal matrices (fruits, cereals and other plants) and many works pointed legumes as a potential source of bioactive compounds [11,12]. Leguminous plants (Leguminosae) have been widely consumed since ancient times and, nowadays, they are globally valued because of their culinary, nutritional and bioactive properties. The relevance of legumes has been demonstrated in many works, since they are a good source of proteins, dietary fibers, complex carbohydrates, phenolic compounds, phytates, saponins, alkaloids, tannins, etc. [13,14,15]. Among all nutrients, proteins from legumes have been the most investigated molecules as source of proteins in human diet, and their consumption has been associated with the prevention of chronic diseases attributable to their bioactive components. Nowadays, a great number of scientific reports evidence the association between the presence of legumes in human diet and the reduction of several chronic diseases risk [11,13,16]. Within plant-based foods, seeds and legumes represent a protein source of great nutritional interest. The protein content in these foods is very high compared to other groups of plant foods, as would be the case of cereals, representing between 17–30% of the edible part in the case of legumes. In addition, proteins of high biological value have been identified within these groups of plant foods, highlighting the proteins of soy [17]. Moreover, within the mentioned functional compounds that can be found in legumes, bioactive proteins and peptides have attracted greater attention. A significant number of whole proteins (e.g., lectins or globulins) and hydrolysates from these species have been studied, as well as renowned peptides such as lunasine or Bowmann-Birk inhibitor from soybean, and several biological properties have been described from these compounds [18,19,20]. The presence of bioactive peptides in legumes can contribute to increase their food protein quality value, adding functional properties to food consumed on a daily basis [2]. For all the above-mentioned, the development of functional ingredients, including bioactive peptides from vegetal sources those can prevent or treat any of the emerging pathologies previously described, is a priority field in food science research.

This work reviews the beneficial effects of different proteins and peptides derived from legumes and updated the main findings of in vitro experimental studies investigating the effects of legumes consumption on non-transmissible chronic diseases, specifically on metabolic syndrome and related disorders. An exhaustive revision of five relevant bioactivities, including four of them directly related to metabolic syndrome: antioxidant, anti-inflammatory, antihypertensive and hypocholesterolemic; and antitumoral bioactivity of proteins, hydrolysates and peptides derived from legumes were carried out. The work was focused on isolation and purification, enzymatic hydrolysis and in vitro gastrointestinal digestion, establishing correlations between proteins, hydrolysates or specific peptides from legumes and biological activities, including potential mechanism of action.

## 2. Materials and Methods

The related scientific literature was reviewed and the studies were identified using Web of Knowledge (https://www.webofscience.com/ (accessed on 1 November 2022)), Scopus (https://www.scopus.com/ (accessed on 1 November 2022)) and PubMed (https://pubmed.ncbi.nlm.nih.gov/ (accessed on 1 November 2022)) databases. Combinations of several search terms such as “legumes”, “peptides”, “hydrolysate/s”, “bioactive”, “in vitro”, “in vivo”, “clinical trial”, “antioxidant”, “anti-inflammatory”, “immune-modulatory”, “microbiota”, “hypocholesterolemic”, “antihypertensive”, “antitumoral”, “antiproliferative”, “hypolipidemic” were applied. After the revision, studies were classified according to the leguminous source, the bioactive fraction obtention method and the experimental model used for the bioactivity evaluation.

## 3. Results

Several studies have been studied the same legumes as a source of proteins those were used to obtain hydrolysates and/or peptides with potential bioactive properties. From all activities, research in this field has been focus on five bioactivities: antioxidant, anti-inflammatory, antihypertensive, hypocholesterolemic and antitumoral. The references commented in the present review were summarized in Table 1, and specific identified peptide sequences were compilated in Table 2.

### 3.1. Antioxidant Activity

Oxidative stress is caused by a disturbance in the balance between free radicals production and antioxidant defenses. It is implicated in several disorders such as cardiovascular and neurodegenerative diseases or cancer, among others. Dietary antioxidants can help to maintain the mentioned balance and reduce oxidative stress. Vegetal sources could be used with this purpose.

Plants and plant extracts have been utilized as natural antioxidants since ancient times, and a relevant percentage of the scientific reports correlated this capacity to their phenolic compounds content [83]. However, these properties could also be caused by the presence of proteins and/or peptides obtained after hydrolysis releasing antioxidant peptide sequences. The interest previously mentioned on searching for antioxidant compounds of natural origin has led many investigations on the use of food proteins as a potential source of peptides with antioxidant properties, due to its low cost, high bioactivity and easy absorption [84]. Although the mechanisms of antioxidant action of food-derived peptides have not been fully elucidated, several studies postulated that these peptides are inhibitors of lipid peroxidation and free radical scavengers [85,86,87]. Moreover, some antioxidant peptides showed to be capable of protecting cells from the damage produced by Reactive Oxygen Species (ROS) by stimulating the expression of genes related to antioxidant defense proteins. Furthermore, some food-derived peptides have demonstrated the ability to diminish free radical formation in human endothelial cells via induction of heme oxygenase-1 and ferritin [88].

Many works have been carried out using different species of legumes with this purpose. All of them agreed in the concept that hydrolysis procedures led to the release of products with higher antioxidant capacity when compared to the native protein. For that matter, the use of commercial enzymes (alcalase, flavorzyme, corolase PP, savinase, neutrase, etc.) in soybean, cowpea, pea, lupin bean, mung bean and African yam bean proteins produced antioxidant hydrolysates with higher capacity of radical scavenging and lipid peroxidation inhibition [20,21,25,36,37,79].

Besides, an increase of the antioxidant power was correlated with a decrease of the molecular size of the obtained peptides, after hydrolysis of chickpea proteins (particularly from 200 to 3000 Da) and African yam bean proteins (particularly <1000 Da) with alcalase. It was also related with the increase of their hydrophobicity as reported by Li, Jiang, Zhang, Mu, & Liu [19] and Ajibola et al. [21], respectively. The mentioned chickpea hydrolysates demonstrated higher free radicals scavenging capacity (up to 85% for DPPH radical, 81% for hydroxyl radical and 69% for superoxide radical) and those obtained from African yam beans showed scavenging activities close to 40% for DPPH and superoxide radicals and 30% for hydroxyl radical when the peptides size was <1000 Da [19,21].

The hydrolysis degree and antioxidant ability of the products could be enhanced under high pressure treatments, such as kidney bean protein hydrolysates described by Al-Ruwaih, Ahmed, Mulla, & Arfat [22]. Although most of the articles did not identify the sequences of the released peptides, Babini et al. [79] isolated and characterized several peptides from pea (TVTSLDLPVLRW and VTSLDLPVLRW) and lupin bean (FVPY) with antioxidant activity (5078, 5477 and 6108 mmol glutathione equivalents/mol peptide, respectively, according to the DPPH radical scavenging assay).

Furthermore, hydrolysis products combining antioxidant peptides and phenolic compounds could also be obtained. In this context, Garcia-Mora et al. [23] investigated pinto beans protein content and used subtilisins (alcalase and savinase) to release some peptides containing widely described antioxidant sequences (FVPH, FVPHYYSK, FYPHYYSKAI, SWN, GHHH) that acted together with phenolic compounds leading to high antioxidant capacities and reaching values within the range of 326–348 Trolox equivalents/g protein (according to the ORAC assay). Moreover, these hydrolysates also exerted antihypertensive and anti-inflammatory properties.

As mentioned before, studies performing in vitro gastrointestinal digestion were also assayed to evaluate not only protein hydrolysis, but also differences in the phenolic compounds profile before and after digestion and, consequently, their antioxidant capacity. These studies were carried out in lentil, black soybean and black turtle bean, showing that the content and the type of individual free phenolic acids were drastically modified by in vitro digestion procedure. These findings suggested further investigation to understand how these changes might affect the digestibility of these legume bioactive compounds and, therefore, their functionality [24].

### 3.2. Anti-Inflammatory Activity

Inflammation is a biological process in response to infection, injury or irritation [89]. Chronic inflammation seems to be associated with different types of diseases, such as arthritis, allergy, cardiovascular diseases and even some types of cancer [90]. The anti-inflammatory effects of numerous plant extracts and isolated compounds rich in antioxidants have already been scientifically demonstrated [91].

Although fewer studies have been developed testing the anti-inflammatory effects of proteins or derived peptides from legumes when compared to other bioactivities, the most widely studied peptide has been lunasin. Vernaza, Dia, de Mejia, & Chang [29] found a significant inhibition on inflammatory markers such as nitric oxide (NO), nitric oxide synthase (iNOS), prostaglandin E2 (PGE2), cyclooxygenase 2 (COX-2), and Tumor Necrosis Factor alpha (TNF- α) in lipopolysaccharide (LPS)-induced RAW 264.7 macrophages using peptides from soybean flour. Moreover, Dia, Wang, Oh, de Lumen, & de Mejia [26] investigated the anti-inflammatory activities of lunasin and lunasin-like peptides from soybean flour. They demonstrated a decrease of the pro-inflammatory responses in RAW 264.7 macrophages for defatted soybean flour, soybean lunasin and lunasin-like peptides [26,30] and, later, they also demonstrated the anti-inflammatory effect of specific peptides such as VPY produced after soybean enzymatic hydrolysis [31]. Moreover, Hernandez-Ledesma, Hsieh & de Lumen [92] concluded that lunasin inhibits TNF-α and Interleukin 6 (IL-6) production in RAW 264.7 macrophages. Furthermore, in LPS-induced human THP-1 macrophages, lunasin exerted anti-inflammatory effects by inhibiting the protein kinase B-mediated (Akt-mediated) Nuclear Factor-κB (NF-κB) pathway [93]. Besides, similar results were obtained with hydrolysates from other species such as pea (using thermolysin) [27], and García-Mora et al. [23] reported anti-inflammatory properties for hydrolysates combining peptides (NEGEAH, DNPIFSDHQ, SGSYFVDGHH) and phenolic compounds obtained from pinto beans using alcalase and savinase.

Lectins can also display either pro- or anti-inflammatory actions depending on the administration route used via lectin domain interaction [94]. Leite et al. [28] found anti-inflammatory activity from purified and characterized lectins in the seeds of butterfly pea (*Clitoria fairchildiana*).

### 3.3. Antihypertensive Activity

Hypertension is a chronic medical condition in which the systemic arterial blood pressure is consistently elevated to at least 140 mm Hg systolic or 90 mm Hg diastolic [95]. Blood pressure is mainly regulated by the renin–angiotensin system (RAS). Renin converts angiotensinogen to angiotensin I that could be activated by conversion to angiotensin II by angiotensin-converting enzyme (ACE). Angiotensin II is a potent vasoconstrictor and rapidly increases the blood pressure. Persistent high blood pressure is a controllable risk factor for cardiovascular diseases such as stroke and heart failure, which are the leading causes of death and disability worldwide [96]. The inhibition of enzymes in the RAS pathway, especially ACE, is regarded to be a potent therapeutic approach in the treatment of hypertension [97], and the most effective drugs to treat hypertension are potent ACE inhibitors.

Regarding functional food research, antihypertensive capacity is one of the most studied bioactivities of proteins and peptides from legumes, and ACE inhibition is one of the most studied mechanisms. Soybean proteins, hydrolysates and peptides have recently attracted much attention on their health benefit for hypertension management and to avoid secondary effects of synthetic drugs. ACE inhibitory activity peptides derived from soybean hydrolysates have been described and, in some cases, with simultaneously antioxidant properties [41,95].

Coscueta et al. hydrolyzed soybean flour proteins with corolase PP to obtain several peptides (IRHFNEGDVLVIPPGVPY, IRHFNEGDVLVIPPGVPYW, IYNFREGDLIAVPTG, VSIIDTNSLENQLDQMPRR, YRAELSEQDIFVIPAG) that behaved as in vitro ACE inhibitors being part of hydrolysates with promising IC_50_ values (reaching 220 and 178 µg/mL when the hydrolysis was carried out at 70 and 90 °C, respectively) [20]. Furthermore, a pentapeptide from the soybean 11 S globulin, which could be released after treatment with protease P, exerted ACE-inhibitory activity (IC_50_ = 1.7 µM) and showed resistance to digestion by gastrointestinal proteases [42]. Potential health benefits of protein hydrolyzed with alcalase from mung bean have been reported since the hydrolysates exerted antihypertensive effect by inhibiting ACE in vitro (IC_50_ = 10–20 µg/mL) [37], and pea protein hydrolysates obtained with alcalase and thermolysin were also studied as an interesting source of antihypertensive peptides. Alcalase-obtained dipeptides (IR, KF, EF) showed in vitro ACE inhibitory activities (IC_50_ = 2.3, 7.2 and 3.0 mM, respectively), as well as thermolysin-obtained ones (IFENNLQN, FEGTVFENG; IC_50_ = 87.5 and 76.8%, respectively). Moreover, FEGTVFENG and LTFPG (both obtained with thermolysin) were able to decrease the systolic blood pressure when they were administered to hypertensive rats [38,39].

The effect of fermentation and in vitro digestion on antihypertensive activity of soybean and pea peptides has also been evaluated, considering as key processes to release potential ACE inhibitory peptides [40,43]. ACE inhibitory peptides from legume proteins were also isolated from fermented pea flour by *Lactobacillus plantarum* strain (KEDDEEEEQGEEE) [40], or through fermentation of soybean with different strains of lactic acid bacteria (HHL, PGTAVFK) [80].

Other varieties of legumes have also showed ACE inhibitory activity: the octapeptide (PVNNPQIH) was released after in vitro digestion of small red beans and exerted this capacity [33]; ACE inhibitory peptides from lentils subjected to high pressure hydrolysis (REQIEELRRL, DLAIPVNRPGQLQSF and DLPVLRWLKL, among others) have also been identified [35], and hydrolysis of mung beans also led to the production of ACE inhibitory hydrolysates [32].

Besides, a study performed in chickpea hydrolysates elucidated the relevance of the presence of methionine and other hydrophobic amino acids for antihypertensive properties, since they were key to exert ACE inhibitory capacity [34], and also the importance of the molecular size, since low molecular weight-peptides were more effective than high molecular weight ones to inhibit ACE [18].

### 3.4. Hypocholesterolemic Activity

As mentioned before, cardiovascular diseases are, together with cancer, one of the major causes of death in developed countries and high levels of total cholesterol and serum low-density lipoprotein (LDL)-cholesterol constitutes one of the main risk factors for both diseases. Cholesterol levels depend on dietary absorption and endogenous synthesis, and, in this sense, many food-based extracts and ingredients have been developed claiming hypocholesterolemic capacities, but the marketed products can only reduce cholesterol absorption without inhibiting cholesterol biosynthesis, suggesting that further research must be carried out [98]. In this context, different peptides from legumes hydrolysates have also shown effectiveness in lowering blood cholesterol levels. Due to this, a significant number of in vitro studies can be found in the literature but also in vivo analysis, being more commonly used than in other biological activities.

During cholesterol endogenous biosynthesis pathway, the 3-hydroxy-3-methylglutaryl coenzyme A reductase (HMGCR) plays a key role and in vitro HMGCR inhibitory activity is frequently tested for potential hypocholesterolemic activity [98]. In fact, Aiello et al. [81] noticed that three peptides (IAVPGEVA, IAVPTGVA and LPY) that derived from soybean protein hydrolysis (with trypsin and pepsin) exerted HMGCR inhibitory activity. The authors also evaluated the potential intestinal absorption of these peptides, detecting both intact compounds and degradation fragments (AVPTGVA, IAVPT, IAVP).

Several works have focused on soybean globulins fractions as potential hypocholesterolemic compounds. For instance, 7 S globulin showed interesting in vitro and in vivo results, being able to reduce total cholesterol, LDL-cholesterol and triglycerides in the plasma and liver of hypercholesterolemic rats by oral administration [47]. Besides, the isolation of the α’ subunit of this protein led to a decrease in plasma lipids and upregulated liver β-VLDL receptors in hypercholesterolemic rats [48]. Moreover, an octapeptide (IAVPGEVA) released from pepsin 11 S globulin hydrolysis, revealed, besides HMGCR inhibitory activity, in vitro bile acid binding capacity, giving rise to the stimulation of serum cholesterol transformation into bile acids [49]. Soybean protein hydrolysis with alcalase also generated a pentapeptide (WGAPS) that displayed in vitro ability to reduce cholesterol content in micelles and, consequently, cholesterol absorption [82].

Other legumes species were evaluated with this purpose, since white lupin seeds protein extracts reduced cholesterolemia in rats and in vitro studies suggested that it could be explained by the stimulation of LDL receptor activity. This effect was observed when a concrete white lupin protein (conglutin γ) was assayed in HepG2, a human liver cancer cell line [45]. Lupin peptides derived after treatment with pepsin, have also showed a positive influence on cholesterol metabolism in Caco-2 cells, a human model of the intestinal epithelial barrier, decreasing the proprotein convertase subtilisin/kexin type 9 (PCSK9) secretion [46]. Moreover, lunasin has also attracted great attention due to its potential hypocholesterolemic capacity. The purposed mechanisms for lunasin are based on two actions: first, the ability of blocking histone acetylation (inhibiting histone H3 tail acetylation), reducing the expression and production of HMGCR; second, the induction of an increase in the expression of the LDL-receptor gene, leading to the cleaning of LDL-cholesterol from bloodstream [99,100]. Furthermore, CPe-III peptide from chickpea (*Cicer arietinum L.*) significantly reduced serum total cholesterol, triglycerides and hepatic triglyceride levels changes induced by a high-fat diet in hyperlipidemic mice [50]. Common bean (*Phaseolus vulgaris L.*) protein hydrolysates showed hypocholesterolemic activity preventing inflammation and dysfunction of vascular endothelium, indicating an adjuvant effect on reducing atherogenic risk, in mice feeding with an atherogenic diet [51].

In vitro simulated human digestion also released peptides from cowpea proteins with hypocholesterolemic activity. The resulted peptides were able to decrease cholesterol solubilization in the micelles and in silico assays suggested that they might inhibit HMGCR by binding and modifying its active site [44].

Besides hypocholesterolemic activity, different hydrolysates obtained from lentil soluble proteins using savinase or subjecting them to gastrointestinal digestion were tested as pancreatic lipase inhibitors since a reduction in lipase activity leads to hypolipidemic effects. However, the obtained hydrolysates exerted lower inhibition capacities than the non-hydrolyzed fraction [101]. Moreover, frequent consumption of legumes could help with the management of obesity by lowering lipid levels [102]. Oseguera-Toledo, Gonzalez de Mejia, Sivaguru, & Amaya-Llano [103] demonstrated the biological in vitro potential of common bean peptides by the inhibition of lipid accumulation in adipocytes cells 3T3-L1.

### 3.5. Antitumoral Activity

Cancer is one of the leading causes of mortality and morbidity in occidental developed countries and its occurrence is increasing largely due to the ageing of the population. Cancer is a collection of related diseases with multifactorial etiology that involved a complex multistep development process of tumor formation caused by the uncontrolled cell proliferation (by evasion from growth suppressors or immune destruction, resistance to cell death, sustained proliferative signaling, etc.) [104]. Besides a severe health problem, cancer is also an economic burden and plant chemicals are a promising line for research on cancer. In this context, proteins, hydrolysates and peptides from legumes have demonstrated potential antitumoral activity.

Soybean hydrolysates produced with thermoase constituted a source of hydrophobic peptides against P388D1 (a mouse monocyte macrophage cell line) proliferation, arresting cell cycle progression at G2/M phases. The primary sequence of the antitumoral peptide was determined as XMLPSYSPY [105]. Soybean proteins were also hydrolyzed by Wang, Rupasinghe, Schuler & de Mejia [58] with pepsin and pancreatin, and hydrolysis procedure led to several peptides (FEITPEKNPQ, IETWNPNNKP, VFDGEL) that were able to inhibit topoisomerase II activity and, therefore, impeding cell proliferation and differentiation in carcinogenesis due to the inactivation of this enzyme. Recently, black soybean proteins were also hydrolyzed with alcalase and a tetrapeptide (L-I/VPK) that was isolated from these hydrolysates showed inhibitory activity against HepG2 (human liver cancer), MCF-7 (human breast cancer) and HeLa (human cervix cancer) cells proliferation [59].

Giron-Calle, Alaiz & Vioque [106] utilized pepsin and pancreatin to obtain chickpea protein hydrolysates that showed inhibitory activity against THP-1 (human leukemia monocytes) cells proliferation. Also in chickpeas, Xue et al. [53] hydrolyzed albumin with Flavorzyme and observed that the generated hydrolysates exerted antitumoral activity in mice that were injected with H-22 (murine hepatic carcinoma) tumor cells.

Moreover, widely described peptides such as lunasin can be found in soybean and other legumes, exerting potent antitumoral activity. Probably, lunasin is one of the most studied bioactive peptides from vegetal sources. It was firstly isolated in 1987 from soybean seeds by ethanolic extraction followed by ion-exchange and reverse-phase chromatography [107]. Its sequence contains 43 amino acids and it is mostly found in soybean, but also in some cereals such as barley and wheat, and pseudocereals such as amaranth and quinoa [108,109,110]. Lunasin is a peptide with a promising potential for the prevention and treatment of tumoral cell proliferation and, although its molecular mechanisms of action in different types or stages of cancers have not been elucidated yet, it behaves as an anti-mitotic and antimutagenic agent, also able to inhibit histones by binding them [111]. One of the main concerns related to this peptide is its ability to reach the target organs. In this context, a relevant number of studies testing their bioavailability have been carried out, pointing out that other plant peptides such as Bowman-Birk inhibitor (BBI) can protect lunasin during digestion by proteases inhibition [112,113,114]. Besides, BBI that can be found in some legumes (particularly in soybean) and cereals has also shown antitumoral activity by itself [55,115]. It has been suggested that the combined action of both peptides seems to be a great alternative to prevent or treat carcinogenesis [2].

Due to the importance of lunasin in antitumoral activity, a scalable procedure of soybean lunasin isolation/purification has also been developed [92] and the bioactivity of the resulted product was confirmed, with similar histone-binding capacity than synthetic lunasin. Furthermore, BBI was also successfully isolated from cowpeas, exhibiting promising antitumoral properties in vitro, such as human osteosarcoma cells growth inhibition and breast cancer cells cytotoxicity [54,55,56].

Moreover, lectins are proteins that can be isolated from plants such as legumes. They are highly specific for carbohydrates that are present in specific cells causing their agglutination and precipitation. Although lectins have been avoided by food technicians through decades because of their antinutritive and toxic character, they have also demonstrated potential antitumoral properties [116].

For instance, lectins from beans and lentils were also able to inhibit different lineages of cancer cells proliferation such as human liver (H3B) and human colon (Caco2) cancer cells [52,57]. Moreover, these lectins can be hydrolyzed with pepsin and pancreatin and the obtained hydrolysates showed high antioxidant capacities [62].

Considering that human digestion might release new fractions or peptides that might exert antitumoral properties, several studies have been performed with proteins from legumes, using simulated digestion conditions considering gastric and intestinal enzymes, chemical environment, temperature, movement, etc. Gonzalez-Montoya, Hernandez-Ledesma, Silvan, Mora-Escobedo, & Martinez-Villaluenga evaluated in vitro digested proteins from soybean and reported the antiproliferative effect of these hydrolysates on human colorectal cancer cells (Caco-2, HT-29 and HCT-116) [61].

### 3.6. Other Biological Activities

In addition to the previously described activities, other capacities were reported to hydrolysates and peptides released from different legumes species. For instance, chelating peptides and hydrolysates have been obtained from soybean, chickpea and beans. This property signifies a great potential since they may be capable of chelating free forms of minerals such as calcium, iron or copper, avoiding ROS generation and the subsequent oxidative events, but also enhancing mineral absorption and bioavailability [62,64]. The works that can be found in the scientific literature highlighted the relevance of high molecular size in chelating abilities [66], but also high histidine content for copper binding and the presence of glutamic and aspartic acids for calcium binding [65,66,68]. Regarding this fact, Zhang, Li, Miao, & Jiang hydrolysed chickpea protein using alcalase to obtain a copper and iron-chelating pentapeptide (NRYHE) [63] and Lv, Bao, Liu, Ren, & Guo identified a soybean peptide released after the enzymatic action of protease M and glutaminase with high affinity to calcium (DEGEQPRPFPFP) [67].

Moreover, antimicrobial aminoacidic sequences have been identified and studied in legume proteins. The importance of these properties is essential for food industry since they can be utilized to prevent or eliminate non-desirable microorganisms not only in foodstuffs, i.e., with a technological purpose, but also in human organism, i.e., with a clinical perspective [69,70]. These antimicrobial peptides can be released carrying out a simulated gastrointestinal digestion, using proteolytic enzymes such as bromelain, trypsin, alcalase, etc. [69,71,72] and also by the action of fermentative microorganisms [70,73]. For instance, HTSKALLDMLKRLGK was an identified peptide released after soybean fermentation by *Bacillus subtilis* that exerted antimicrobial activity against several pathogens [73].

Some bioactive peptides from legumes have also been described as immunomodulatory agents, showing that they are able to positively modify the immune response. Maintaining the correct function of immune system is a key factor for human health and it can be linked to beneficial effects towards the prevention of metabolic syndrome, since some inflammatory processes can be avoided as non-specific immune mechanisms. In this sense, Tsuruki et al. found that an immunostimulating peptide derived from soybean beta-conglycinin, was an *N*-formyl-Met-Leu-Phe (fMLP) agonist [76]. In addition, some lectins, a family of carbohydrate-binding proteins which occur in nearly all food matrices but the highest amounts are found in legumes, have showed immunomodulatory effects to certain pathological conditions, such as cancer and infections [117].

Another interesting field of action of bioactive peptides is related to glucose homeostasis, and peptides derived from legumes can also influence glucose metabolism. Nowadays, peptide drugs used for the treatment of diabetes are being obtained in large scale via synthesis or by using the recombinant DNA technology, but these drugs are comparatively more expensive than small molecules and are not affordable to most of the patients. Therefore, research on bioactive peptides from natural origin is remarkably increasing. Antidiabetic legumes peptides are still under exploration and few bioactive peptides have been reported. Until now, bioactive sequences from soybeans, chickpeas, lupin or beans with antidiabetic properties have been investigated and they showed alpha-glucosidase, alpha-amylase and/or dipeptidyl peptidase IV (DPP-IV) inhibitory activities [74,75,77,78].

## 4. Conclusions

The importance of legumes goes beyond their nutritional value, and they constitute a recurring source of compounds that exert biological properties. Among them, proteins are the most widely studied molecules and many works were compiled in this revision regarding enzymatic hydrolysis of these leguminous proteins, in vitro assays to evaluate the biological activity, simulation of gastrointestinal digestion, and, in some cases, purification and identification of specific peptides. Peas, chickpeas, cowpeas, lupin and particularly soybean and beans are the most utilized pulses in these studies. The obtained peptide products have demonstrated interesting bioactivities such as antioxidant, anti-inflammatory, antihypertensive, hypocholesterolemic and antitumoral. However, most of them (except for hypocholesterolemic) are mainly tested using cell cultures and other in vitro protocols; only a few works validated these promising properties in animal models and no clinical trials were found in the literature focused on specific fractions, hydrolysates or peptides. In vivo studies using experimental models and validation in human subjects should be the future perspective of researchers in the field of legumes bioactivities to achieve the development of novel functional foods based on these molecules. Moreover, these in vivo studies are necessary to demonstrate or provide information on the bioavailability of these biologically active peptides. Therefore, the multiple properties of legume-derived peptides after hydrolysis suggest that they could target several symptoms of metabolic syndrome. For all of this, the approach of a new research line from a multifunctional point of view could be very interesting in order to develop novel plant-based foods, taking advantage of the nutritional and sustainable benefits that legumes present, to improve our health and that of the planet.

## Figures and Tables

**Table 1:** Summary of the published works describing the obtention of bioactive protein/peptides fractions from different species of legumes.

Activity	Leguminous Source	Obtention of Bioactive Fraction	Experimental Model	Reference
Antioxidant	Bean	Alcalase hydrolysis	In vitro	[21,22]
		Alcalase/Savinase hydrolysis	In vitro	[23]
		In vitro digestion	In vitro	[24]
	Chickpea	Alcalase hydrolysis	In vitro	[19]
	Cowpea	Alcalase/Flavourzyme/pepsin-pancreatin	In vitro	[25]
	Lentil	In vitro digestion	In vitro	[24]
	Lupin	Bacterial and Alcalase/Neutrase/Flavourzyme hydrolysis	In vitro In vitro	[26]
	Pea			
	Soybean	Corolase PP hydrolysis	In vitro	[20]
		In vitro digestion	In vitro	[24]
Anti-inflammatory	Bean	Alcalase/Savinase hydrolysis	In vitro	[23]
	Pea	Thermolysin hydrolysis	In vitro/in vivo (mice)/ex vivo	[27]
		Lectin isolation	In vivo (rats)	[28]
	Soybean	Alcalase hydrolysis	In vitro	[29]
		Lunasin isolation	In vitro	[26]
			In vitro	[30]
		Specific peptides isolation	In vivo (mice)	[31]
Antihypertensive	Bean	Alcalase hydrolysis	In vitro	[32]
		In vitro digestion	In vitro	[33]
	Ckickpea	Alcalase hydrolysis	In vitro	[34]
	Lentil	Alcalase/Protamex/Savinase/Corolase 7089 hydrolysis	In vitro	[35]
	Mung bean	Alcalase hydrolysis	In vitro	[36]
			In vitro	[37]
	Pea	Thermolysin hydrolysis	In vitro/in vivo (rats)	[38]
		Alcalase hydrolysis	In vitro	[39]
		In vitro digestion/fermentation	In vitro	[40]
	Soybean	Pepsin/pancreatin hydrolysis	In vitro	[41]
		Corolase PP hydrolysis	In vitro	[20]
		Protease P/trypsin/chymotrpypsin	In vitro	[42]
		Fermentation	In vitro	[43]
Hypocholesterolemic	Cowpea	In vitro digestion	In vitro	[44]
	Lupin	Total protein extraction	In vivo (rats)	[45]
		Pepsin/Trypsin hydrolysis	In vitro	[46]
	Soybean	7S globulin isolation	In vitro/in vivo (rats)	[47]
			In vivo (rats)	[48]
		Pepsin hydrolysis	In vitro	[49]
	Chickpea	CPE-III peptide	In vivo (mice)	[50]
		Pepsin/pancreatin hydrolysis		[51]
Antitumoral	Bean	Lectin isolation	In vitro	[52]
	Chickpea	Flavorzyme hydrolysis	In vivo (mice)	[53]
	Cowpea	BBI isolation	In vitro	[54,55,56]
	Lentil	Lectin isolation	In vitro	[57]
	Soybean	Pepsin/pancreatin hydrolysis	In vitro	[58]
		Alcalase hydrolysis	In vitro	[59]
		Lunasin isolation	In vitro	[60]
		In vitro digestion	In vitro	[61]
Mineral-chelating	Bean	Pepsin + pancreatin hydrolysis	In vitro	[62]
	Chickpea	Alcalase hydrolysis	In vitro	[63]
		Pepsin + pancreatin hydrolysis	In vitro	[64]
		Alcalase/flavourzyme hydrolysis	In vitro	[65]
	Soybean	Neutrase/flavourzyme hydrolysis	In vitro	[66]
		Protease M + glutaminase hydrolysis	In vitro	[67]
		Protease M + deamidase hydrolysis	In vitro	[68]
Antimicrobial	Bean	Alcalase hydrolysis	In vitro	[69]
	Bitter bean	Boiling + *L. fermentum* fermentation	In vitro/in silico	[70]
	Butterfly pea	Bromelain/trypsin hydrolysis	In vitro	[71]
	Soybean	Gastrointestinal digestion	In vitro	[72]
		*B. subtilis* fermentation	In vitro	[73]
Immune-modulatory	Bean	Pepsin/pancreatin/hydrolysis	In vitro	[74]
	Black bean	Alcalase hydrolysis	In vitro	[75]
	Soybean	Germinated	In vitro	[76]
Antidiabetic	Soybean	Pepsin/pancreatin/hydrolysis	In vitro	[77]
	Bean	Pepsin/pancreatin/hydrolysis	In vitro	[78]

**Table 2:** Compilation of the peptide sequences with biological activities identified in different species of legumes.

Peptide Sequence	Leguminous Source	Biological Activity	Reference
HTSKALLDMLKRLGK	Pea	Antioxidant	[79]
VTSLDLPVLRW			
FVPY	Lupin		
FVPH	Pinto bean		[23]
FVPHYYSK			
FYPHYYSKAI			
SWN			
GHHH			
NEGEAH		Anti-inflammatory	
DNPIFSDHQ			
SGSYFVDGHH			
IRHFNEGDVLVIPPGVPY	Soybean	Antihypertensive	[20]
IRHFNEGDVLVIPPGVPYW			
IYNFREGDLIAVPTG			
VSIIDTNSLENQLDQMPRR			
YRAELSEQDIFVIPAG			
IR	Pea		[39]
KF			
EF			
IFENNLQN			
FEGTVFENG			[38,39]
LTFPG			[38]
KEDDEEEEQGEEE			[40]
HHL			[80]
PGTAVFK			
PVNNPQIH	Small red bean		[33]
REQIEELRRL	Lentil		[57]
DLAIPVNRPGQLQSF			
IAVPGEVA	Soybean	Hypocholesterolemic	[49,81]
IAVPTGVA			[81]
LPY			[81]
WGAPS			[82]
FEITPEKNPQ		Antitumoral	[58]
IETWNPNNKP			[58]
VFDGEL			[58]
NRYHE		Mineral-chelating	[63]
DEGEQPRPFPFP			[67]
HTSKALLDMLKRLGK			[73]

## Data Availability

Not applicable.
